# PET/MR Imaging of a Lung Metastasis Model of Clear Cell Renal Cell Carcinoma with (2*S*,4*R*)-4-[^18^F]Fluoroglutamine

**DOI:** 10.1007/s11307-022-01747-9

**Published:** 2022-06-22

**Authors:** Alyssa C. Pollard, Vincenzo Paolillo, Bhasker Radaram, Sarah Qureshy, Li Li, Tapati Maity, Lei Wang, Md. Nasir Uddin, Christopher G. Wood, Jose A. Karam, Mark D. Pagel, David Piwnica-Worms, Steven W. Millward, Natalie Wall Fowlkes, William Norton, Brian J. Engel, Federica Pisaneschi, Niki M. Zacharias

**Affiliations:** 1 Department of Cancer Systems Imaging, MD Anderson Cancer Center, 1881 East Rd, Houston, TX 77054 USA; 2 Department of Chemistry, Rice University, 6100 Main St, Houston, TX 77005 USA; 3 Center for Advanced Biomedical Imaging (CABI), MD Anderson Cancer Center, Cyclotron Radiochemistry Facility1881 East Rd, Houston, TX 77054 USA; 4 Department of Urology, MD Anderson Cancer Center, 1515 Holcombe Blvd, Houston, TX 77030 USA; 5 Department of Translational Molecular Pathology, MD Anderson Cancer Center, 1515 Holcombe Blvd, Houston, TX 77030 USA; 6 Department of Veterinary Medicine and Surgery, MD Anderson Cancer Center, 1515 Holcombe Blvd, Houston, TX 77030 USA

**Keywords:** PET/MRI, Glutaminolysis, [^18^F]fluoroglutamine, ASCT2, Kidney cancer, Renal cell carcinoma lung metastasis

## Abstract

**Purpose:**

Metabolic reprogramming plays an important role in the tumorigenesis of clear cell renal cell carcinoma (ccRCC). Currently, positron emission tomography (PET) reporters are not used clinically to visualize altered glutamine metabolism in ccRCC, which greatly hinders detection, staging, and real-time therapeutic assessment. We sought to determine if (2*S*,4*R*)-4-[^18^F]fluoroglutamine ([^18^F]FGln) could be used to interrogate altered glutamine metabolism in ccRCC lesions in the lung.

**Procedures:**

We generated a novel ccRCC lung lesion model using the ccRCC cell line UMRC3 stably transfected with GFP and luciferase constructs. This cell line was used for characterization of [^18^F]FGln uptake and retention by transport analysis in cell culture and by PET/MRI (magnetic resonance imaging) in animal models. Tumor growth in animal models was monitored using bioluminescence (BLI) and MRI. After necropsy, UMRC3 tumor growth in lung tissue was verified by fluorescence imaging and histology.

**Results:**

In UMRC3 cells, [^18^F]FGln cell uptake was twofold higher than cell uptake in normal kidney HEK293 cells. Tracer cell uptake was reduced by 60–90% in the presence of excess glutamine in the media and by 20–50% upon treatment with V-9302, an inhibitor of the major glutamine transporter alanine-serine-cysteine transporter 2 (ASCT2). Furthermore, in UMRC3 cells, [^18^F]FGln cell uptake was reduced by siRNA knockdown of ASCT2 to levels obtained by the addition of excess exogenous glutamine. Conversely, [^18^F]FGln cellular uptake was increased in the presence of the glutaminase inhibitor CB-839. Using simultaneous PET/MRI for visualization, retention of [^18^F]FGln *in vivo* in ccRCC lung tumors was 1.5-fold greater than normal lung tissue and twofold greater than muscle. In ccRCC lung tumors, [^18^F]FGln retention did not change significantly upon treatment with CB-839.

**Conclusions:**

We report one of the first direct orthotopic mouse models of ccRCC lung lesions. Using PET/MR imaging, lung tumors were easily discerned from normal tissue. Higher uptake of [^18^F]FGln was observed in a ccRCC cell line and lung lesions compared to HEK293 cells and normal lung tissue, respectively. [^18^F]FGln cell uptake was modulated by exogenous glutamine, V-9302, siRNA knockdown of ASCT2, and CB-839. Interestingly, in a pilot therapeutic study with CB-839, we observed no difference in treated tumors relative to untreated controls. This was in contrast with cellular studies, where CB-839 increased glutamine uptake.

**Supplementary Information:**

The online version contains supplementary material available at 10.1007/s11307-022-01747-9.

## Introduction

Kidney cancer is one of the 10 most common cancers in the USA, and approximately 90% of kidney malignancies are diagnosed as renal cell carcinoma (RCC) [1]. Clear cell renal cell carcinoma (ccRCC) is the most common RCC subtype (≈ 70%). Even after nephrectomy, distant ccRCC metastases occur frequently, with the lungs being the most common site of metastasis [1, 2]. ccRCC has recurrence rates of up to 30% with the highest risk of recurrence occurring within the first 3 years following surgery. However, RCC metastases have been found up to 37 years after surgical resection [3].

The most common image-based assessment following radical nephrectomy is computerized tomography (CT) of the thorax and abdomen. This typically occurs 6 months post-surgery with follow-up imaging every 12 months for 3 years [4]. Current CT imaging protocols cannot easily determine the malignancy of indeterminate pulmonary nodules (IPNs). IPNs can only be determined as malignant by observing increased nodule size over time, which requires multiple imaging sessions and can delay treatment. Patient care would greatly benefit from new imaging techniques that discriminate between benign and malignant IPNs.

Many positron emission tomography (PET) agents are designed to interrogate specific metabolic pathways upregulated in cancer, but the most common PET agent used in oncology, 2-[^18^F]fluoro-2-deoxy-D-glucose ([^18^F]FDG), is unreliable in RCC [5–7]. In a study that imaged 50 ccRCC lesions by PET/CT, only 34% (17/50) were [^18^F]FDG-avid, and maximum standardized uptake values (SUVmax) ranged from 1.55 to 3.83 [5]. Two meta-analyses using [^18^F]FDG-PET in RCC recently evaluated the diagnostic performance for detecting metastatic or recurrent lesions in patients [7, 8]. Both revealed that the sensitivity of [^18^F]FDG-PET can be quite low due to the low metabolic activity of pulmonary lesions, and [^18^F]FDG tumor uptake is very dependent on the size of the lesion with lesions smaller than 5 mm being hard to accurately evaluate. Therefore, there is an unmet need for PET radiotracers capable of identifying the metabolic changes associated with malignant IPNs.

Metabolic reprogramming is an important factor in ccRCC carcinogenesis, and approximately 90% of ccRCC lesions have an inactive von Hippel Lindau (*VHL)* gene [1]. Inactive VHL protein leads to increases in the stability of hypoxia inducible factor 1α (HIF1α) and 2α (HIF2α) and upregulation of multiple metabolic proteins [9, 10]. An increased influx of glutamine has been observed in cancers with high HIF expression or hypoxic tumors [11]. Glutaminolysis is a multibranch pathway upregulated in cancer that generates energy, supports biomass production, and mediates the redox balance by providing glutamate for the biosynthesis of glutathione. Alanine-serine-cysteine transporter 2 (ASCT2), also named SLC1A5, is a major glutamine transporter overexpressed in many cancers [12]. In ccRCC, high ASCT2 expression is associated with lower overall survival and is identified as an independent prognostic factor [13]. A novel ASCT2 inhibitor named V-9302 was recently reported and shown to inhibit tumor growth in colorectal animal models [12].

After transport into a cell, glutamine can be metabolized through several metabolic pathways [11]. In the mitochondria, glutaminase (GLS) converts glutamine to glutamate, which is metabolized to α-ketoglutarate, a key metabolite in the citric acid cycle. In a recent clinical trial, monotherapy with the glutaminase inhibitor CB-839 led to stable disease in over 50% of patients with relapsed/refractory RCC [14]. When used in combination with everolimus, an mTOR kinase inhibitor used as late-line therapy for advanced RCC; CB-839 treatment led to an 8.5-month progression free survival benefit compared to everolimus alone [15]. These clinical data illustrate the importance of glutamine metabolism in RCC and the promise of GLS inhibition for its treatment. Although targeting glutamine metabolism in RCC is a promising therapeutic strategy, there are no imaging techniques available to identify the patients most likely to respond to GLS inhibition and no methods to rapidly assess response to GLS inhibitors.

(2*S*,4*R*)-4-[^18^F]Fluoroglutamine ([^18^F]FGln) is a PET tracer that is transported into cells via the same transporters as endogenous glutamine, mainly ASCT2 [16, 17]. [^18^F]FGln has been found to undergo minimal metabolism in the cell [18], and it is retained in the intracellular compartment upon ASCT2-mediated transport. Therefore, [^18^F]FGln presents a unique opportunity to interrogate active glutamine import in RCC tumors. [^18^F]FGln has been used to image gliomas in humans [17] and has been evaluated preclinically in multiple cancer animal models including colorectal, lung, breast, and primary RCC [12, 16, 18, 19]. The present study reports on the use of [^18^F]FGln in ccRCC cell lines and as a PET radiotracer in a mouse model of ccRCC lung metastasis. Our data reveal that [^18^F]FGln uptake is dependent on ASCT2 expression in cell culture, and this uptake can be reduced by extracellular glutamine or inhibition of glutamine transport. In animal models, PET/Magnetic Resonance Imaging (MRI) with [^18^F]FGln was superior to PET/CT in detecting lung metastases.

## Materials and Methods

### Cell Uptake of [^18^F]FGln

All cells were grown in low glucose Dulbecco's Modified Eagle Medium (DMEM) without glutamine (Millipore Sigma-Aldrich, D5546, St. Louis, MO) supplemented with 10% (v/v) fetal bovine serum (Millipore Sigma-Aldrich, F0926), 300 mg/ml streptomycin, 100 μg/ml penicillin (Corning, 30–002-CI, Corning, NY), and 1 × non-essential amino acids (Corning, 25–025-CI). Prior to each experiment, a fresh 200 mM stock of glutamine (Millipore Sigma-Aldrich, G7513) was added to the media to a final concentration of 2 mM. In CB-839 experiments, 10 mM stock of CB-839 (Selleckchem, S7655, Houston, TX) was added to the media to a final concentration of 3 µM. Cells from a T175 flask were detached and re-suspended in low glucose DMEM with 2 mM glutamine and grown for 48 to 72 h prior to performing the experiment. For V-9302 experiments, cells were incubated with 50 µM of a V-9302 solution (Selleckchem, S8818) in low glucose DMEM media with 2 mM glutamine one hour prior to harvesting. Cells were harvested for [^18^F]FGln uptake experiments by trypsinization (0.25% (v/v) trypsin with 2.21 mM EDTA, Millipore Sigma-Aldrich, T4049), isolated by centrifugation, suspended in phosphate buffer (Corning, 21–040-CV), counted, and then subdivided into 1 million cells per microcentrifuge tube. Trypan blue staining was used to determine cell viability, and cells were counted using a Countess automated cell counter (ThermoFisher, Waltham, MA). Cells were centrifuged at 1500 × g for 2 min at 4 °C, and the phosphate buffer was removed.

For 1-h uptake experiments, cells were resuspended in 1 mL of media and equilibrated in a 37 °C humidified incubator in an atmosphere containing 5% (v/v) CO_2_. Cells were suspended in serum-free low glucose DMEM media without glutamine, with 20 mM glutamine, with 50 µM V-9302, or with 3 µM CB-839. Then, 50 μCi of [^18^F]FGln were added, and cells were incubated for 1 h at 37 °C with mixing every 20 min. After incubation, cells were centrifuged at 400 × g for 3 min and washed 3 × with 1 mL ice cold PBS. After washing, cells were transferred into 3-mL polystyrene tubes, and the radioactivity was counted using a Wizard 2 2480 Gamma Counter (Perkin Elmer Inc, Waltham, MA). For 2-min uptake experiments, cells were resuspended in 0.1 mL of media described above and equilibrated in a 37 °C Eppendorf Thermomixer heated shaker (Eppendorf AG, Hamburg, Germany). Then, 5 μCi of [^18^F]FGln were added, and cells were incubated with shaking for 2 min. Uptake was quenched by the addition of 1 mL ice-cold PBS. Cells were washed and counted as above.

Cell uptake was expressed as a concentration ratio defined as follows: (CPM_Cells_/1 × 10^6^ cells)/(CPM_Input_/mL), where CPM = counts per minute. Cell uptake results were graphed using GraphPad Prism 7 (GraphPad Software, San Diego, CA, USA). Statistical analysis was performed using a one-way ANOVA with Dunnett’s multiple comparisons test, p-value < 0 0.05 (*), < 0.01 (**), < 0.001 (***).

### siRNA Knockdown

UMRC3-LUC-GFP were cultured as described in the Supplementary Materials. 30% confluent cells were subcultured to a new plate/dish a day before transfection. siRNAs were transfected into cells according to the manufacturer’s instructions (Millipore Sigma-Aldrich). Briefly, 1 nM siRNA was transfected using 1 μl MISSION siRNA Transfection Reagent (Millipore Sigma-Aldrich, S1482). All siRNAs were bought from Millipore Sigma-Aldrich. Three different siRNA pairs targeting the ASCT2 sequence were used. Sequences were ASCT2-1 (5’-GUCAGCAGCCUUUCGCUCA-3’, 5’-UGAGCGAAAGGCUGCUGAC-3’), ASCT2-2 (5’-CCAAGCACAUCAGCCGUUU-3’, 5’-AAACGGCUGAUGUGCUUGG-3’), and ASCT2-3 (5’-GAGGAUGUGGGUUUACUCU-3’, 5’-AGAGUAAACCCACAUCCUC-3’). A negative control contained only transfection reagent. Cells were harvested 24 h post-transfection. ASCT2-3 was used for ASCT2 knockdown in the [^18^F]FGln cellular uptake experiment. [^18^F]FGln uptake and Western blot experiments were performed 48 h after transfection.

### Western Blot

Proteins were extracted using a radioimmunoprecipitation assay (RIPA) buffer (Thermo Fisher, 89900) with proteinase inhibitor (Thermo Fisher, 862495), and quantification of total protein was performed using the DC™ protein assay regents (BioRad, Hercules, CA). 20–40 μg of protein was then resolved on 10% Mini-PROTEAN TGX gels (BioRad, 4561034) using SDS-PAGE running buffer and transferred onto polyvinylidene difluoride (PVDF) (BioRad, 162074). Blots were blocked with 5% milk (Biorad, 170640XTU) for one hour and then incubated with a mouse anti-ASCT2 antibody (1:1000 dilution, Cell Signaling, 8057S, Danvers, MA) overnight at 4 °C. Blots were visualized using anti-rabbit HRP-linked secondary antibody (1:5000 dilution, Cell Signaling, 7074S). Protein loading was determined by incubating blot with a mouse anti-β-actin HRP-linked antibody (1:5000 dilution, Cell Signaling, 12262S) or a mouse anti-Histone-H3 HRP-linked antibody (1:5000 dilution, Cell Signaling, 12648P) for one hour at room temperature. Immobilon western HRP substrate (Millipore Sigma-Aldrich, P90720) was used for visualization. Densitometry was measured using Image J (NIH) or Image Studio Lite (LI-COR Biosciences). For ASCT2 Western blots, cell lysates were treated with PNGase F (BioRad, P0711S) then incubated for 10 min at 50 $^\circ$ C following the manufacturer’s one-step protocol.

### Mouse Models

All mouse experiments were approved by the University of Texas MD Anderson IACUC. Subcutaneous tumor models were generated in five male NSG (NOD-scid) mice by subcutaneous injection of 100 µl of phosphate buffered saline containing 5 million SN12C cells. For the orthotopic lung tumor model, mice were injected in groups of 10 to 15 using both female and male Swiss nude mice. Both NSG and nude mice were purchased from MD Anderson Experimental Radiation Oncology. Our method of orthotopic injection was similar to other reported procedures [20, 21]. Mice were anesthetized using 3% isoflurane and placed in the right lateral decubitus position. Sustained-release buprenorphine was administered subcutaneously, and a small incision was made caudal to the foreleg to open the skin and allow for visualization. A 0.5-ml syringe with a permanently attached 27G needle (BD, 305,620, Franklin Lakes, NJ) was used to inject 50 µl of a 1:1 mixture of Matrigel (Corning 354,262) and phosphate-buffered saline containing 3 million UMRC3-LUC-GFP cells into each mouse. The needle was advanced 5–7 mm into the thorax and removed after injection. The skin was closed using wound clips, and the mouse was observed until recovery. The wound clips were removed ten days after surgery.

### Fluorescence Necropsy Imaging

Mice were anesthetized with carbon dioxide and then euthanized by exsanguination via cardiac stick. The sternum was removed, and the tongue, larynx, trachea, thyroid, parathyroids, esophagus, thymus, lungs, and heart were removed *en masse*. The tissue was imaged using a dissection stereomicroscope (Leica Biosystems, MZ16A, Buffalo Grove, IL) with a fluorescent light source. Images were captured at 0.5 × magnification in brightfield and with a GFP filter using Leica LAS software. After imaging, lungs were slowly insufflated with 10% neutral buffered formalin by passing a blunt needle attached to a syringe through the larynx for histology. Formalin-Fixed Paraffin-Embedded (FFPE) blocks were generated from lung tissue. Histological slides were cut and stained with hematoxylin and eosin. Slides were then scanned using Aperio AT2 total slide scanner (Leica Biosystems, AT2) and reviewed by a veterinary pathologist (N.W.F.).

### PET/CT Imaging, Reconstruction, and Analysis

All scans were performed using a Bruker Albira PET/CT preclinical imaging system. Mice were fasted for 6 h, anesthetized with 2% isoflurane using oxygen as a carrier, and then injected intravenously with ~ 200 μCi [^18^F]FGln. The actual injected dose was calculated by measuring the pre- and post-injection activity in the syringe using a CRC-15R dose calibrator (Capintec, Inc., Florham Park, NJ, USA). CT parameters were as follows: 400 μA, 45 kV, 250 projections. Scatter, randoms, decay, and attenuation corrections were applied through the Albira software. For dynamic PET experiments, the radiotracer was injected with the mouse in the scanner, and the images were acquired for an hour followed by a 5 min CT. For static PET experiments, animals were allowed to recover from anesthesia after tracer injection before being put back under anesthesia prior to scanning. Static PET scans were acquired over 20 min. CT images were reconstructed using a Filtered Back Projection algorithm, and PET images were reconstructed using a Maximum Likelihood Expectation–Maximum algorithm with 12 iterations. Volumes of interest (VOI), representative images, and time–activity curves (TACs) were generated using PMOD v3.610 software. Injected doses were used to calculate percent of injected dose per cubic centimeter of tissue (%ID/cc). Errors in the averaged %ID/cc were reported as standard deviation. Figures were prepared using GraphPad Prism 8 (GraphPad Software, San Diego, CA, USA).

### MR Imaging

Mice were imaged using a 7 T horizontal-bore MR scanner with a 30-cm bore and Avance HD architecture (Bruker, Billerica, MA) equipped with 20 cm fixed gradients. Mice were placed on a heated sled fitted with a nose cone allowing the mice to be anesthetized with 2% isoflurane using oxygen as a carrier throughout the imaging experiment. The breathing rate was monitored throughout the experiment. The following sequences were used for all MR imaging sessions: a localizer for placement, 2D coronal rapid acquisition with relaxation enhancement (RARE) (TE = 57 ms, TR = 2200 ms, 40 × 30 mm FOV, 3 averages, 19 slices, 0.75 mm thickness with 0.25 mm gap between slices, 256 × 192 matrix), 2D axial RARE (TE = 39 ms, TR = 2200 ms, FOV 40 × 30 mm, 3 averages, 26 slices, 0.75 mm thickness with 0.25 mm gap between slices, 256 × 192 matrix), and 3D RARE acquired in the coronal view (TE = 57 ms, TR = 1500 ms, 45 × 32 × 20 mm FOV, 1 average, 32 slices, 20 mm slice thickness, 257 × 32 × 20 matrix, 175 × 175 × 625 mm resolution). Respiratory gating was used in all sequences except for the initial localizer. For PET/MRI studies, the PET image was obtained simultaneously with the 3D RARE acquisition.

### PET/MR Imaging, Reconstruction, and Analysis

All scans were performed on a Bruker 7 T MRI (Bruker, Billerica, MA) with a Cubresa NuPET^TM^ insert (Cubresa, Inc., Winnipeg, MB). Dynamic PET images were acquired for 1 h immediately after radiotracer injection with concurrent MR image acquisition. Mice were fasted for 6 h, anesthetized with 2% isoflurane using oxygen as a carrier, and then injected intravenously with ~ 200 μCi [^18^F]FGln in the PET/MRI scanner. The actual injected dose was calculated by measuring the pre- and post-injection activity in the syringe using a CRC-15R dose calibrator (Capintec, Inc., Florham Park, NJ, USA). Scatter, randoms, and decay corrections were applied through the Cubresa software. For dynamic analysis, data were binned into 30 s time frames for the first 5 min and 5 min frames for the next 55 min. To produce static images, data were binned over the entire hour-long scan. Reconstructions were performed using an Ordered Subset Maximum a Posteriori One Step Late algorithm with 8 iterations and 4 subsets, and images were quantified using a Quantification Calibration Factor for [^18^F]fluorine. PET/MR image registration was manually performed using the 3D RARE MR image and inviCRO VivoQuant (inviCRO, LLC, Boston, MA) software. VOIs, representative images, and TACs were also generated using inviCRO VivoQuant software. Injected doses were used to calculate percent of injected dose per cubic centimeter of tissue (%ID/cc). Errors in the averaged %ID/cc were reported as standard deviation. Figures were prepared using GraphPad Prism 8.

### CB-839 and Vehicle *in vivo* Treatment and Image Analysis

Nude mice were inoculated in a similar manner to previously described. Tumor growth was monitored by BLI in 16 mice throughout the experiment. Mice were imaged with [^18^F]FGln on a Bruker 7 T MRI (Bruker, Billerica, MA) with a Cubresa NuPET^TM^ insert (Cubresa, Inc., Winnipeg, MB). Dynamic PET images were acquired for 40 min immediately after radiotracer injection with concurrent MR image acquisition. The mice were then subdivided into vehicle and CB-839 treated. One cohort (8 mice) received a daily dose [22] of CB-839 (MedChemExpress, catalog number HY-12248 Monmouth Junction, NJ) (200 mg/kg dose of CB-839). To increase our solubility of the compound, we suspended CB-839 at 10 mg/ml dilution in 30% PEG (MedChemExpress, HY-Y0873), 10% ethanol (Millipore Sigma-Aldrich, E7023), 60% water (Sterile Water, Millipore Sigma-Aldrich, 4.86505.0500), 0.5% Tween 80 (Millipore Sigma-Aldrich, P4780), and methylcellulose (Millipore Sigma-Aldrich, M0430) [23, 24], and mice were orally gavaged with this CB-839 solution daily for 15 days. The other cohort (8 mice) were orally gavaged with the vehicle daily for 15 days. Three mice died during the treatment phase (2 in the vehicle and 1 in the CB-839 cohort). The remaining mice were imaged with [^18^F]FGln on the 14th day or 15th day of treatment. In total, 11 mice were imaged before and during treatment with [^18^F]Gln. Mice were then treated with CB-839 or vehicle until euthanized, and necropsy was performed (1 to 2 days post-imaging). Gross pathology and GFP detection were performed in a similar manner as described above using the fluorescence dissection stereomicroscope. Fluorescent lung tissue was dissected and flash frozen with liquid nitrogen. The flash frozen tissue was then used for metabolomics and Western blot analysis.

### Metabolomics

To determine the relative abundance of polar metabolites in tissue samples, extracts were prepared and analyzed by ultra-high-resolution mass spectrometry (HRMS). For tissue samples, approximately 10 to 20 mg of tissue was pulverized on liquid nitrogen then homogenized with Precellys Tissue Homogenizer. Metabolites were extracted using 1 mL ice-cold 80/20 (v/v) methanol/water. Extracts were centrifuged at 17,000 g for 5 min at 4 °C, and supernatants were transferred to clean tubes followed by evaporation to dryness under nitrogen.

Dried extracts were reconstituted in deionized water, and 10 μL was injected for metabolite analysis by ion chromatography (IC)-MS. IC mobile phase A (MPA; weak) was water, and mobile phase B (MPB; strong) was water containing 100 mM KOH. A Thermo Scientific Dionex ICS-5000 + system was used which included a Thermo IonPac AS11 column (4 µm particle size, 250 × 2 mm) with the column compartment kept at 30 °C. The autosampler tray was chilled to 4 °C. The mobile phase flow rate was 360 µL/min, and the gradient elution program was: 0–5 min, 1% MPB; 5–25 min, 1–35% MPB; 25–39 min, 35–99% MPB; 39–49 min, 99% MPB; and 49–50 min, 99–1% MPB. The total run time was 50 min. To assist with the desolvation for better sensitivity, methanol was delivered by an external pump and combined with the eluent via a low dead volume mixing tee. Data were acquired using a Thermo Orbitrap Fusion Tribrid Mass Spectrometer under ESI negative ionization mode. Then, the same samples were injected for glutamine detection by liquid chromatography (LC)-MS. LC mobile phase A (MPA) was 95/5 (v/v) water/acetonitrile containing 20 mM ammonium acetate and 20 mM ammonium hydroxide (pH ~ 9), and mobile phase B (MPB) was acetonitrile. The Thermo Vanquish LC system was used and included a Xbridge BEH Amide column (3.5 µm particle size, 100 × 4.6 mm) with the column compartment kept at 30 °C. The autosampler tray was chilled to 4 °C. The mobile phase flow rate was 300 µL/min, and the gradient elution program was: 0–3 min, 85% MPB; 3–10 min, 85–30% MPB; 10–20 min, 30–2% MPB; 20–25 min, 2% MPB; and 26–30 min, 2–85% MPB. The total run time was 30 min. Data were acquired using a Thermo Orbitrap Fusion Tribrid Mass Spectrometer under ESI positive ionization mode at a resolution of 240,000. Raw data files were imported to Thermo Trace Finder software for final analysis. The relative abundance of each metabolite was normalized by tissue weight.

## Results

### Synthesis of [^18^F]FGln

We reliably produced [^18^F]FGln with a 8.5 ± 2.9% (n = 7) radiochemical yield using a GE TRACERlab XF2N (Supplemental Fig. 1), following the standard protocol published by Zhang et al*.* [25]. [^18^F]FGln was obtained with a 99% radiochemical purity and 98% optical purity.

### ASCT2 Expression Correlates with [^18^F]FGln Uptake

**Fig. 1:**
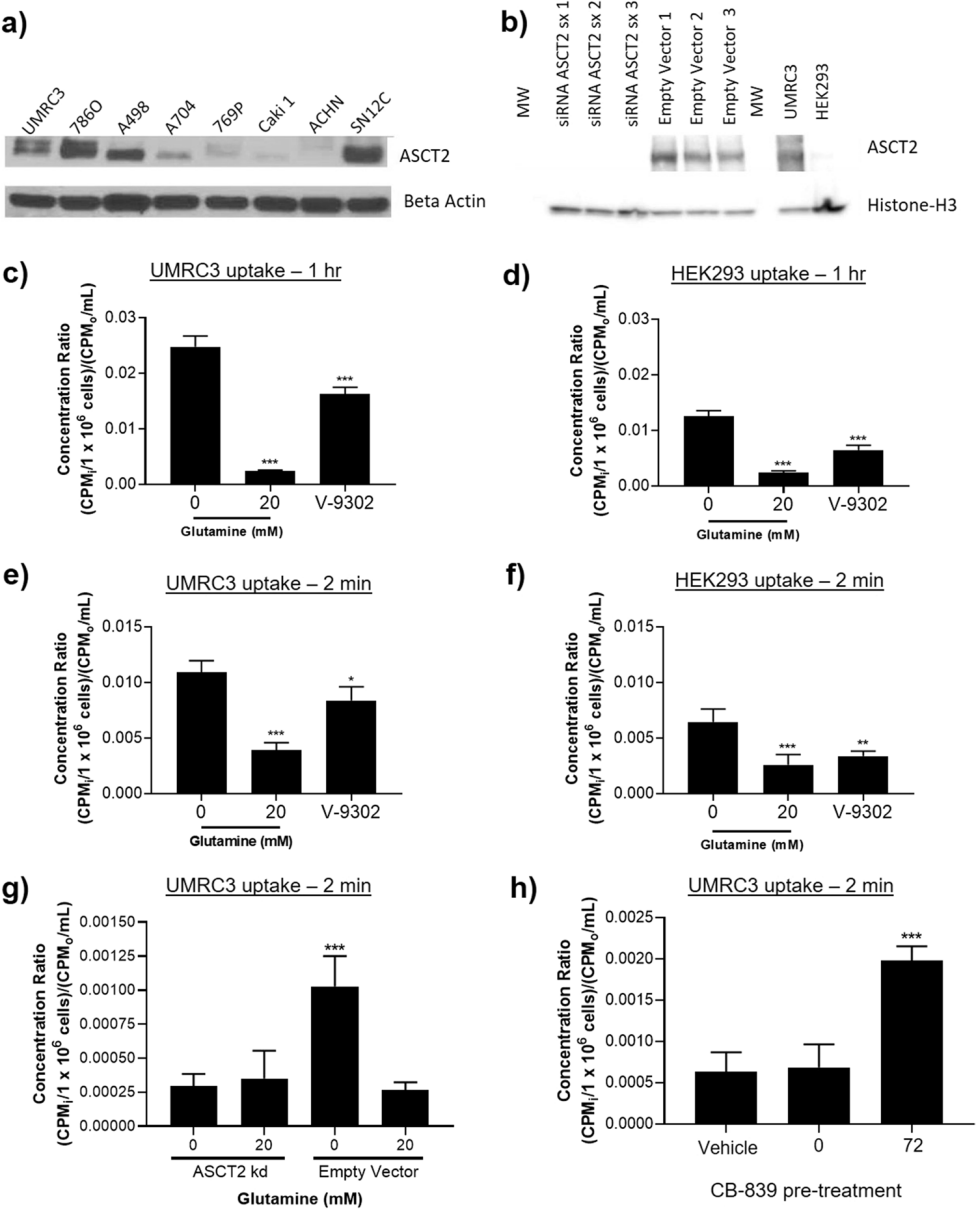
[^18^F]FGln uptake was dependent on ASCT2 expression*.* (**a**) ASCT2 expression was observed in multiple ccRCC cell lines. (**b**) Higher ASCT2 expression was observed in UMRC3 cells compared to HEK293 cells. This expression reduced in UMRC3 cells using siRNA. 1 h [^18^F]FGln uptake was higher in UMRC3 (**c**) than HEK293 (**d**) cells. For both cell types, addition of 50 μM V-9302 or 20 mM glutamine reduced uptake. Similar observations were made with 2-min uptake in UMRC3 (**e**) and HEK293 (**f**) cells. (**g**) siRNA-knockdown of ASCT2 reduced [^18^F]FGln uptake to the level achieved by 20 mM glutamine. (**h**) [^18^F]FGln uptake was increased with pre-treatment of UMRC3 cells with 3 µM CB-839 for 72 h. This increase was not observed when CB-839 was added to media just prior to the uptake experiment (0 h). All uptake experiments were analyzed using a one-way ANOVA with Dunnett’s multiple comparisons test, *** *p* < 0.001, ** *p* < 0.01, and * *p* < 0.05

ASCT2 is differentially expressed in ccRCC cell lines (Fig. 1a). The cell line UMRC3, derived directly from the primary kidney tumor of a patient with metastatic ccRCC, showed higher expression than the normal kidney cell line HEK293 (Fig. 1b) and was amenable to siRNA knockdown of ASCT2. As a VHL mutant cell line, the UMRC3 cell line [26] is thought to be glutamine-dependent. UMRC3 cell injection into a nude or SCID mouse led to tumor formation and metastasis [27]. This cell line was used in all cellular and orthotopic *in vivo* experiments.

[^18^F]FGln uptake in cells was performed by incubating one million cells for 2 min (transporter limited) or 1 h (steady state) with [^18^F]FGln, washing with cold PBS, and measuring cell-associated radioactivity on a gamma counter. Steady-state levels in UMRC3 cells showed tracer uptake was blocked by the addition of exogenous glutamine in the culture media (Fig. 1c). UMRC3 tracer uptake was twofold higher than non-cancerous HEK293 cells (Fig. 1c vs. Figure 1d). These trends were maintained with 2 min uptake experiments, albeit with lower total uptake (Fig. 1e-f). In each case, the addition of 50 µM V-9302, an ASCT2 inhibitor [12], significantly decreased [^18^F]FGln uptake by 20–50%, and addition of exogenous glutamine to the cell culture media significantly reduced uptake by 60–90%. In the UMRC3 cell line, [^18^F]FGln uptake was also reduced with siRNA knockdown of ASCT2 to the level obtained by the addition of exogenous glutamine (Fig. 1g). This confirmed the contribution of this transporter toward glutamine uptake. In contrast, an increase in [^18^F]FGln uptake was detected when UMRC3 cells were treated with 3 µM CB-839 for 72 h prior to uptake experiments (Fig. 1h). In addition, we observed higher expression of ASCT2 in UMRC3 cells treated for 65 h with 3 μM CB-839 compared to the vehicle (Supplemental Fig. 3a). These results were in agreement with similar [^18^F]FGln uptake studies [18, 19].

### Growth of ccRCC Lung Metastases Monitored by BLI

**Fig. 2:**
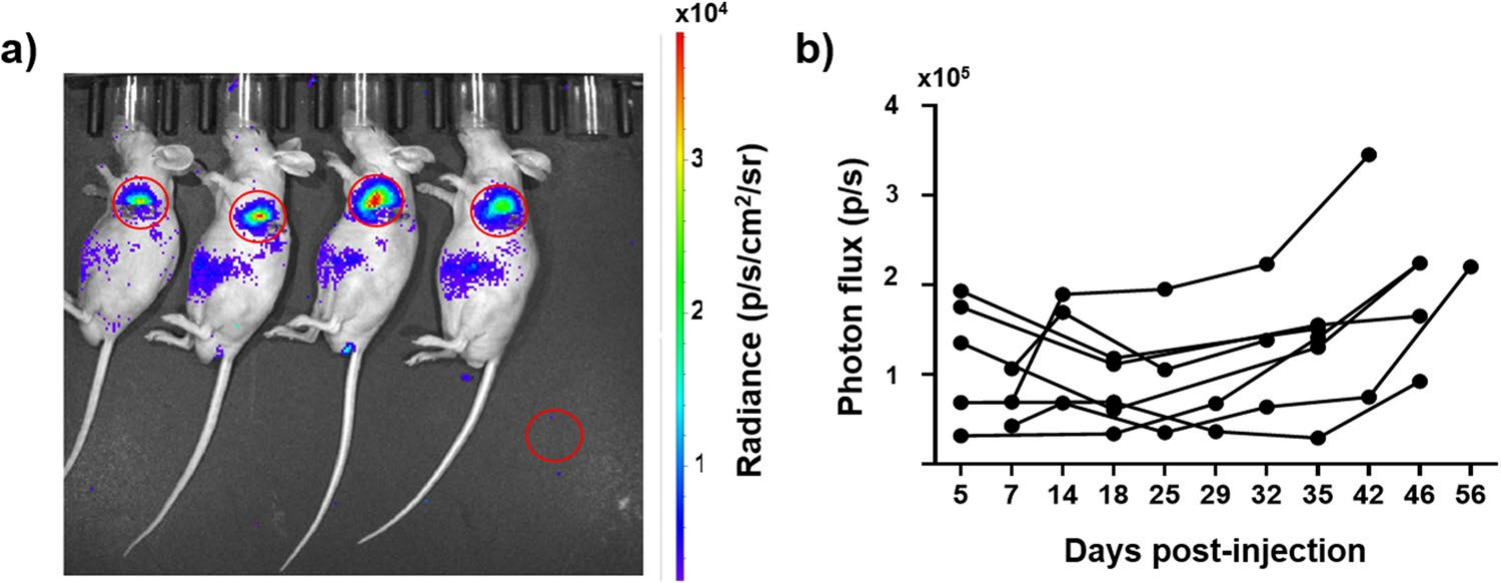
Tumor growth in UMRC3 lung tumor-bearing mice measured by BLI*.* (**a**) Representative BLI of UMRC3 tumor-bearing mice. ROIs (2 cm diameter, red circle) were drawn over the BLI observed in the lung and in the lower right corner of each image to determine background. (**b**) Photon flux  measured in the left lung of 8 UMRC3 tumor-bearing mice are plotted over time. The photon flux is the BLI signal of the lung ROI minus the background ROI

Lung tumors were generated by injecting 3 million UMRC3 cells expressing luciferase and GFP into the lower left lung of nude mice [20, 21]. Tumor formation and growth were monitored using BLI (Fig. 2a). We observed an engraftment rate ranging from 50 to 87% for different cohorts, and BLI was observed five days post-injection. Regions of interest (ROIs) were drawn over the left lung and normalized to a background ROI (Fig. 2b). BLI signal remained constant or increased for all animals over the 1–2 month imaging duration.

### Tumor Location Determined by MRI

**Fig. 3:**
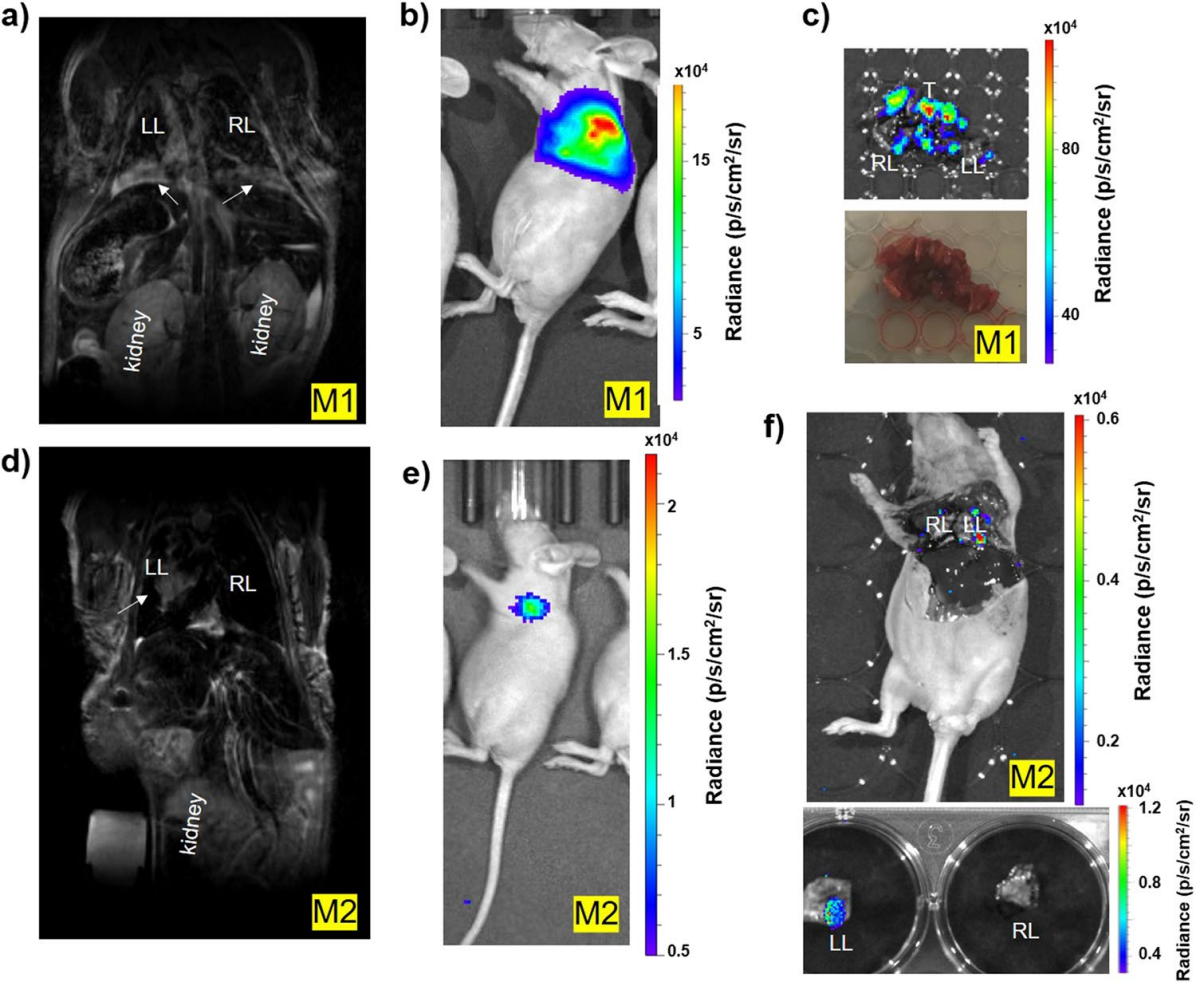
MRI and BLI of UMRC3 tumor-bearing mice*.* (**a**) Representative MR image of a UMRC3 tumor-bearing mouse with tumor growth in the pleural effusion of the lung. The tumor was observed as a bright area in both the right (RL) and left lung (LL) lining (marked by white arrows). (**b**) BLI of the same mouse imaged by MRI in Fig. 3a prior to euthanasia. (**c**) Ex vivo BLI of right (RL) and left (LL) lung tissue of mouse imaged in Fig. 3a and b. BLI was observed in lung tissue and solid tissue (T) in the mediastinum. (**d**) Representative MR image of a UMRC3 tumor-bearing mouse with apparent solid tumor growth within the left lung (LL) (marked by white arrow). (**e**) BLI of the same mouse imaged by MR in Fig. 3d prior to euthanasia. (**f**) Ex vivo BLI of left (LL) and right lung (RL) of mouse imaged in Fig. 3d and e. High BLI signal was observed in lower left corner of the left lung

**Fig. 4:**
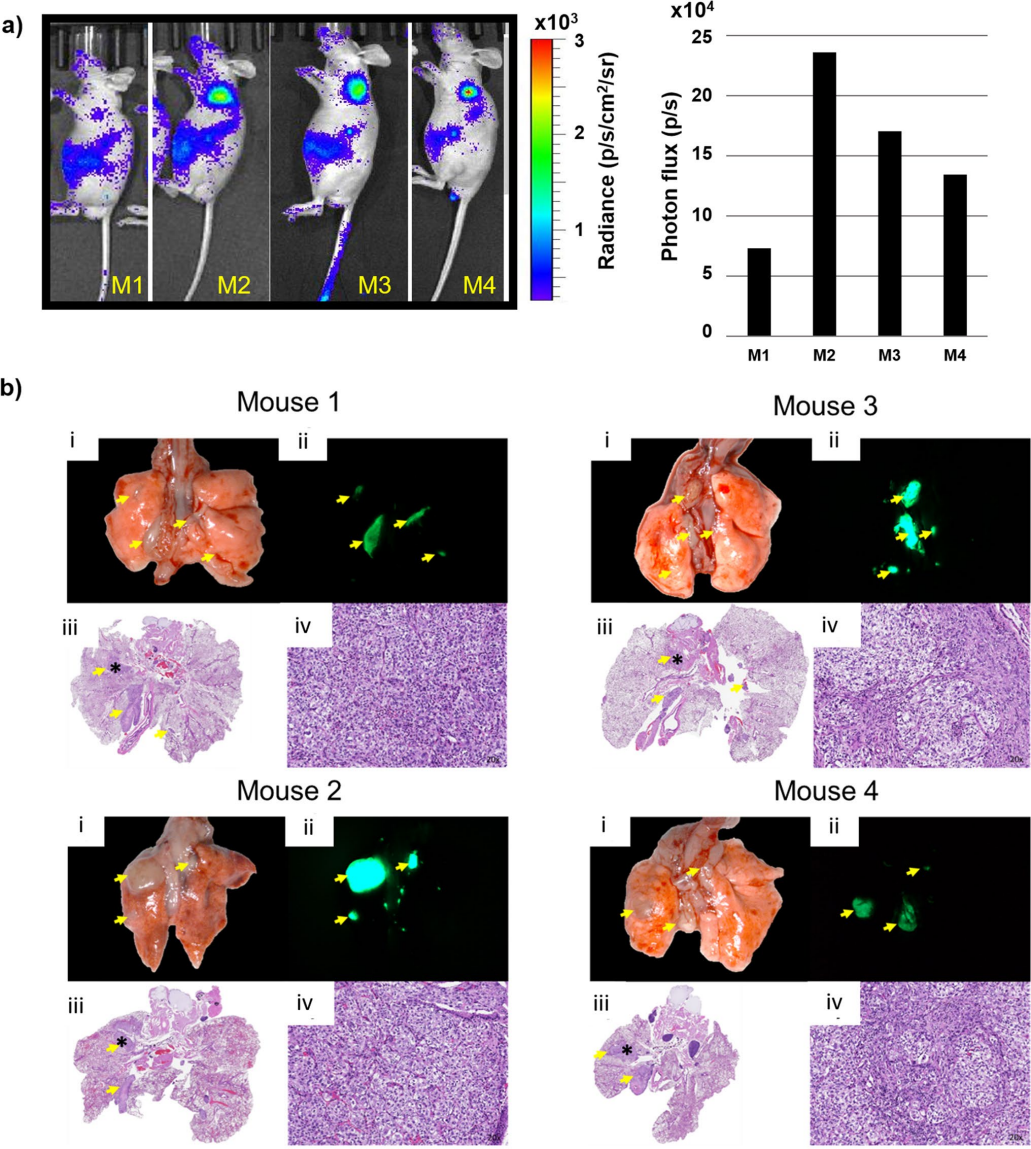
BLI and GFP fluorescence observed both in left and right lungs of mice injected with UMRC3 cells expressing luciferase and GFP. (**a**) BLI of mice taken one day prior to necropsy and photon flux of ROI drawn over lung (diameter 2 cm) of each mouse given in bar graph. (**b**) Gross pathology, GFP expression, and histopathology of UMRC3 tumors (yellow arrows) from the same mice. (**i**) Brightfield gross pathology of tissue from mice bearing UMRC3 tumors. (**ii**) Fluorescence image of the same tissue illustrating the GFP expression. (**iii**) Brightfield scan of H&E slides of lung specimens at 1 × amplification. Asterisk area is amplified in (**iv**) by 20 × with tumor cells clearly visible

Tumor growth was easily observed using 2D and 3D RARE acquisitions on a 7 T Bruker MR scanner. Based on the MR images, UMRC3 cells grew both in the lung and in the pleural effusion of the lung (Fig. 3), which is a common area of RCC metastatic growth. Prior to euthanasia, UMRC3 tumor-bearing animals were injected with luciferin, optically imaged (Fig. 3b and e), and the lungs were removed to observe BLI directly in the lung tissue (Fig. 3c and f). Despite UMRC3 tumor cells being engrafted into the left lung, most animals had tumor growth in both the right and left lungs, and often in the mediastinum. This engraftment in the contralateral lung demonstrates the metastatic potential of this ccRCC cell line. In another cohort of UMRC3 mice, we were able to validate similar engraftment in both the left and right lungs 21 days post-injection using a fluorescence dissecting stereomicroscope after necropsy (Fig. 4b). Intact lungs were removed from euthanized mice and imaged using GFP fluorescence. In all 4 UMRC3 lung tumor-bearing mice, tumor growth was observed both in the left and right lungs. This tumor growth was further confirmed with histology (Fig. 4b). BLI was directly correlated with the level of fluorescence observed in each mouse.

### [^18^F]FGln Uptake in Mouse Models

Initial PET/CT studies were performed on animals bearing subcutaneous SN12C tumors. ccRCC cell line SN12C had previously been used in [^18^F]FGln uptake experiments [19]. PET/CT allowed for easy observation of [^18^F]FGln uptake in SN12C subcutaneous tumors and confirmed the known biodistribution of the PET tracer. As with other small molecules, [^18^F]FGln is cleared primarily through the renal system (Supplemental Fig. 2). Dynamic imaging showed average tumor-to-muscle ratios (T/M) of 1.2 at 20 min post-injection and 1.3 at 40 min post-injection. Similar T/M values were obtained with static imaging at 1 and 2 h post-injection (Supplemental Fig. 2b).

**Fig. 5:**
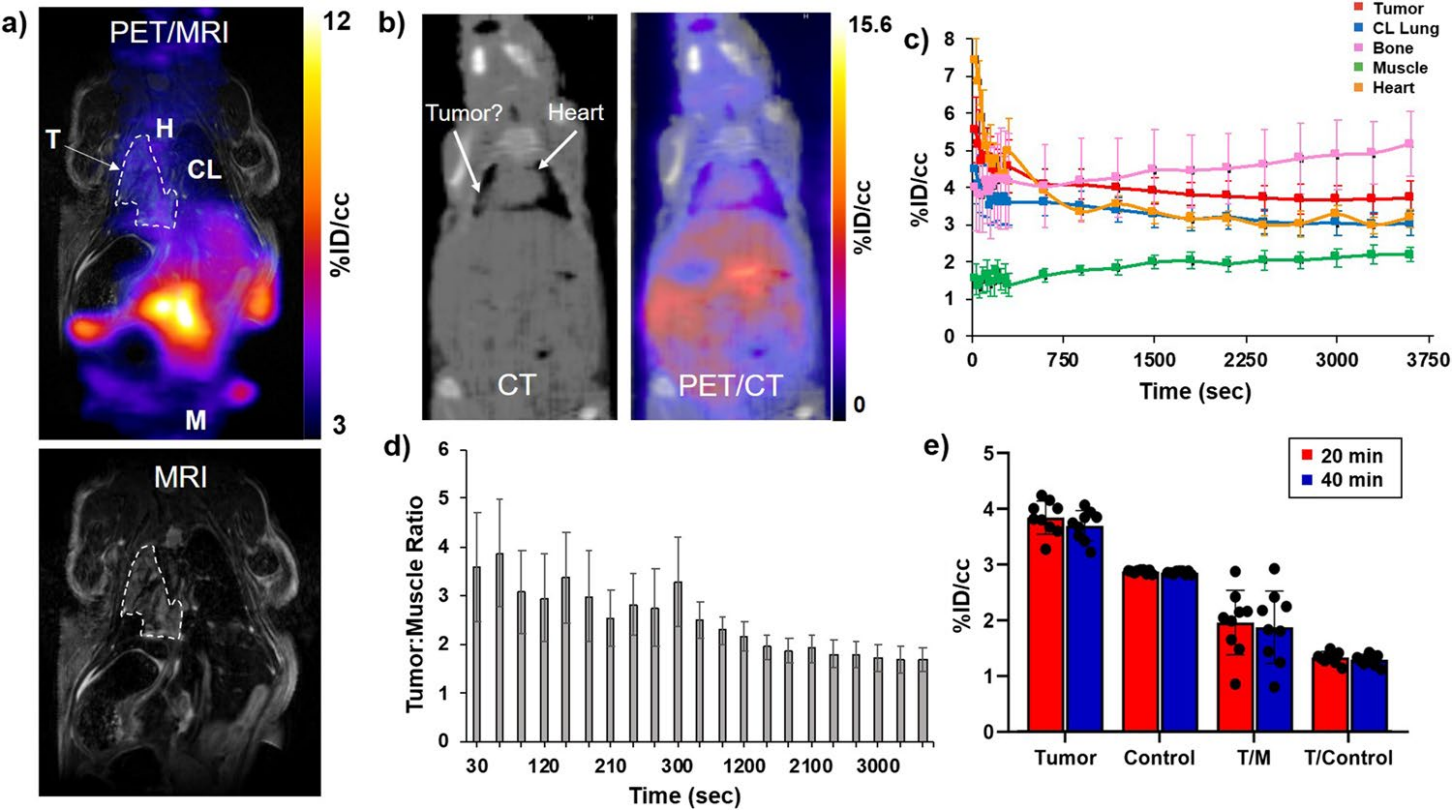
Quantification of [^18^F]FGln uptake in UMRC3 lung tumor-bearing mice and healthy control mice by PET/MR*.* (**a**) Representative [^18^F]FGln static PET image overlaid on a MR image of a UMRC3 tumor-bearing mouse. Areas depicting where VOIs of tumor (T), muscle (M), heart (H), and contralateral lung (CL) were drawn are labeled. Tumor VOI is shown by a white dotted line. (**b**) Representative [^18^F]FGln static PET image overlaid on a CT image of a UMRC3 tumor-bearing mouse. It was impossible to distinguish tumor from heart due to the poor soft tissue contrast of the CT image. (**c**) Time-activity curve showed the average uptake of the tracer in the tumor, contralateral lung, muscle, bone, and heart of the UMRC3 tumor-bearing mouse in Fig. 5a expressed as %ID/cc. Error bars represent the standard deviation within each VOI. (**d**) Tumor-to-muscle ratios (T/M) observed in the UMRC3 tumor-bearing mouse in Fig. 5a over time. Error bars represent the standard deviation within each VOI. (**e**) Plot of tracer uptake in %ID/cc in the lung of tumor-bearing mice (Tumor) and healthy mice (Control) measured 20 min (red) and 40 min (blue) post-injection. T/M of tumor-bearing mice and tumor-to-control mouse lung ratio (T/Control) are also shown in %ID/cc. Each data point represents an individual mouse; colored columns represent the average; error bars represent the standard deviation of the mean

In the orthotopic UMRC3 lung tumor model, PET/CT imaging did not provide enough contrast to allow discrimination of tumors from normal tissue (Fig. 5b). Instead, a 7 T Bruker MR equipped with a Cubresa NuPET^TM^ insert (PET/MRI system) provided superior visualization of the orthotopic lung tumor model. UMRC3 lung tumor-bearing mice were imaged dynamically for 1 h to identify the imaging timepoint with the lowest non-specific uptake. In the MR images, the tumor could be clearly discriminated from normal tissue (Figs. 5a, 3a, and d). In contrast, it was difficult to distinguish tumor from normal tissue such as the heart with CT (Fig. 5b). Tracer uptake was measured in the heart, contralateral lung, tumor, bones, and muscle and reported as %ID/cc. TACs showed higher uptake of [^18^F]FGln in the tumor compared to muscle or contralateral lung at each time point (Fig. 5e). T/M decreased over time from 3 to 2 (Fig. 5d). Based on these TACs, 40 min post-injection was identified as the optimal imaging time point. A similar optimum time window for tumor uptake and blood clearance has been observed in other [^18^F]FGln animal experiments [18]. Because tumor engraftment was often observed in both lungs of UMRC3 tumor-bearing mice, tumor-to-contralateral lung ratios did not accurately reflect the uptake of the tracer compared to healthy tissue. To better understand the tumor-to-healthy lung ratio, uptake of [^18^F]FGln in the lung of tumor-bearing mice was compared with uptake in the lung of healthy mice. This comparison was done by placing the tumor VOI from each tumor-bearing mouse into the left lung of a set of 5 healthy, non-tumor-bearing mice. Uptake of the tracer in the healthy lung was measured for each mouse and averaged to give a unique control for each tumor-bearing mouse. These measurements are labeled as control in Fig. 5e and were used to calculate tumor-to-control (T/C) ratios. Individual measurements taken from each tumor-bearing mouse 20 and 40 min post-tracer injection are shown in Fig. 5e. We observed a median T/M of approximately 2 and T/C of approximately 1.5, validating the usefulness of [^18^F]FGln-PET/MRI to assess renal cell carcinoma lung lesions.

**Fig. 6:**
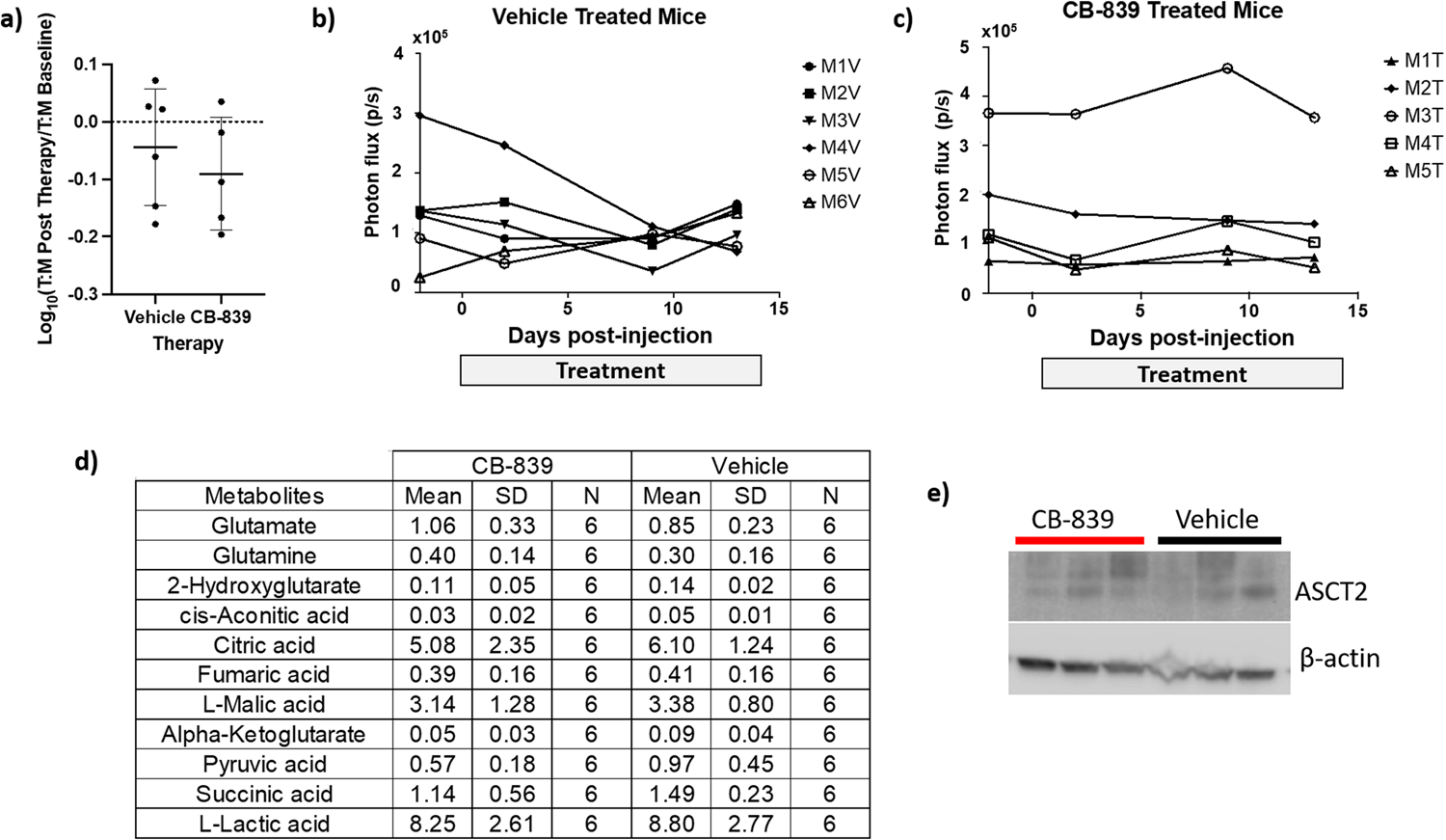
BLI and [^18^F]FGln uptake with glutaminase inhibition. (**a**) The log_10_ of [^18^F]FGln tumor:muscle uptake ratio 14 to 15 days during therapy (%ID/cc) divided by baseline [^18^F]FGln tumor:muscle uptake ratio pre-therapy (%ID/cc) for vehicle and CB-839-treated mice. Each data point represents an individual mouse; bold black line represents the average; error bars show the standard deviation within the dataset; and dotted black line marks a value of 0 (or no change between pre- and post-treatment). A decrease in [^18^F]FGln uptake was observed in CB-839-treated mice compared to the vehicle cohort, but a Student’s two-tailed *t*-test showed no statistical difference between the two datasets (*p* > 0.05). (**b**) Quantification of BLI of each individual mouse plotted prior to therapy and during therapy. A statistically significant difference in BLI was not observed between vehicle (**b**) versus the CB-839 treatment cohort (**c**). (**d**) Table illustrating the relative abundance of multiple metabolites determined by ion chromatography–mass spectrometry (IC-MS) in fluorescent lung tumor tissue treated with CB-839 or vehicle. The mean, standard deviation (SD), and the number of tissues analyzed are given. No difference is observed in glutamine or glutamate levels. (**e**) Western blot of three fluorescent lung tumor tissue samples from CB-839 and vehicle-treated mice. No difference in ASCT2 expression is observed

To determine if [^18^F]FGln uptake would increase after CB-839 treatment *in vivo*, mice with BLI-confirmed UMRC3-GFP-LUC lung tumors were treated with either 200 mg/kg of CB-839 or vehicle daily for 15 days. Mice were imaged with [^18^F]FGln PET/MRI prior to therapy and 14 to 15 days after the start of treatment. While uptake did slightly decrease after treatment for both cohorts, there were no significant differences in post-treatment [^18^F]FGln uptake in the CB-839 or vehicle-treated cohorts (Fig. 6a). Mice were also imaged by BLI 2 days prior to treatment and 2 days, 9 days, and 13 days after beginning treatment (Fig. 6b and c). We did not observe a significant change in BLI prior to treatment versus 13 days post-treatment in either cohort. The bright-field and fluorescent images of the intact lungs for each mouse are shown in Supplemental Fig. 4. The fluorescent tissue was isolated, flash-frozen, and used for metabolite and western blot analysis. The relative abundance of metabolites in each tissue was determined by IC-MS. Similar abundance of all metabolites including glutamine and glutamate was observed in both the CB-839 and vehicle-treated tissues (Fig. 6d). In addition, similar expression levels of ASCT2 were observed in CB-839 and vehicle-treated lysates (Fig. 6e). The difference in glutamine uptake following CB-839 treatment in cells versus animal models could be due to a higher expression of ASCT2 in 2D cultured UMRC3 cells versus UMRC3-derived mouse xenograft tumors. (Supplemental Fig. 3).

## Discussion

[^18^F]FDG PET is the gold standard for cancer diagnosis and staging and is used to identify tissues with high glucose uptake. However, kidney cancer is not [^18^F]FDG avid, and therefore, [^18^F]FDG is not used diagnostically in this tumor type. In ccRCC, new mechanism-based PET tracers are necessary to guide clinical decisions. [^18^F]FGln reports on the expression of glutamine transporters such as ASCT2, which are overexpressed in ccRCC [13]. In this study, the [^18^F]FGln mechanism of action was validated in UMRC3 cellular cultures and a mouse UMRC3 lung lesion model. In 2D cultured UMRC3 cells, higher [^18^F]FGln uptake was observed with respect to normal kidney cells (HEK293) at 2 min and 1 h post-incubation, which suggests a higher influx of the tracer (2 min) and higher retention at steady state (1 h). Cellular uptake was reduced by addition of glutamine to the media.

Cellular uptake of [^18^F]FGln was reduced by knockdown of ASCT2 using siRNA and treatment with the ASCT2 inhibitor V-9302, although this reduction was not as great as the reduction observed with glutamine in media. The observed difference in reduction of [^18^F]FGln uptake after V-9302 treatment compared to the addition of glutamine in the media may be due to off-target effects of V-9302 [28] or compensatory mechanisms. For example, fourteen transporters have been identified that can transport glutamine into a cell, and compensation through the upregulation of one transporter has been observed in cells when another is downregulated [29–31]. The massive redundancy of transporters responsible for glutamine uptake underscores the importance of this metabolite and the challenge of blocking its uptake in tumors. All these data suggest that [^18^F]FGln uptake is a read-out of the expression of ASCT2 in ccRCC but does not provide absolute proof.

Most RCC lung metastasis models are generated from direct injection of RENCA or other RCC cells [32, 33] into the kidney of mice. We report one of the first direct orthotopic mouse models of ccRCC lung lesions. This model was generated by engrafting UMRC3 cells, transfected with luciferase and GFP, into the lung of Swiss nude mice. Tumor growth was monitored by BLI and MRI and confirmed using a fluorescence dissecting stereomicroscope after necropsy and histology. Even though cells were injected into the left lung, tumor growth was observed in both the left and right lung, illustrating the highly metastatic nature of the UMRC3 cell line.

We initially sought to visualize UMRC3-lung lesions by PET/CT, but CT could not provide enough contrast in the model to distinguish tumor from healthy tissues. Using PET/MRI, we were able to easily visualize the lung lesions by MRI and register the PET image to the MR image. [^18^F]FGln PET provided visualization of UMRC3 lung lesions at 20 to 40 min post-tracer injection with a tumor-to-muscle ratio of approximately 2 and tumor-to-healthy lung ratio of 1.5. The high metastatic nature of this cell line prevented the use of the contralateral lung as a control. However, using the lung of healthy mice as a control provided a rigorous and unbiased quantification of [^18^F]FGln uptake.

Next, we sought to analyze [^18^F]FGln uptake modulation upon intervention. Glutamine transport inhibition is a promising therapeutic area, but currently, V-9302 is still only used preclinically [12]. Clinically, glutaminolysis inhibition is mainly focused on the use of glutaminase inhibitors, such as CB-839. One of our aims was to evaluate whether [^18^F]FGln uptake could be used as an indication of glutaminase inhibition, so we analyzed [^18^F]FGln modulation upon CB-839 treatment in cellular culture and mouse models.

In our UMRC3 cellular model, we observed that [^18^F]FGln uptake increased after a 72-h incubation with the glutaminase inhibitor CB-839. It is worth noting that transformation of glutamine to glutamate, catalyzed by glutaminase, is not directly recapitulated by [^18^F]FGln retention because once [^18^F]FGln is transported inside the tumor cell by ASCT2, it does not enter the glutaminolysis pathway. In mice, minimal incorporation (< 10%) of radioactivity into proteins is observed in tumor tissue with [^18^F]FGln, and the primary species found in the tumor and the blood is [^18^F]FGln [18]. However, overexpression of ASCT2, on which [^18^F]FGln directly reports, can be a biomarker for increased glutamine metabolism, and we observed an increase in ASCT2 expression in UMRC3 cells treated with CB-839 by Western blot. Upon blockage of glutaminolysis by the selective inhibitor CB-839, tumor cells can respond by upregulating glutamine transporters, thereby increasing [^18^F]FGln uptake.

Higher glutamine uptake was observed *in vivo* in triple negative breast cancer and orthotopic kidney cancer animal models after CB-839 treatment [18, 19]. However, in our own studies, CB-839 treatment *in vivo* showed the opposite trend, and [^18^F]FGln uptake decreased slightly, albeit below the level of statistical significance. Although we cannot fully explain why enhanced [^18^F]FGln uptake was not observed *in vivo* with CB-839 treatment, it is worth noting that ccRCC lesions in the lung may behave differently than in the kidney, where an increase in [^18^F]FGln uptake was observed using a similar treatment regime [19]. Lung tissue is well perfused and experiences a high partial pressure of oxygen (pO_2_). As such, metabolism in ccRCC lesions in the lungs could be significantly altered compared to the corresponding primary tumor in the hypoxic kidney environment. Moreover, mammalian cell cultures have high metabolic plasticity [34], and the high dependence on glutamine in cancer cells could be associated with 2D tissue culture growth, which is reduced when grown in mouse models. We also observe higher expression of ASCT2 in our UMRC3 cell lysates compared to the lysate of UMRC3 lung tumor tissue. Perhaps more importantly, no statistical difference was observed with BLI in tumor mass reduction in CB-839 versus vehicle-treated mice. In addition, no qualitative differences in fluorescence necropsy images were detected, and no difference in relative abundance of glutamine was observed with IC-MS. These data suggest that the UMRC3 lung lesions do not respond to CB-839 treatment, and a lack of change in [^18^F]FGln uptake could act as a negative predictor of treatment outcome. However, a larger animal study would be needed to confirm, and this is outside the scope of the present study.

Recently, two patients with lung lesions, one RCC metastasis lung lesion and one non-small cell lung cancer lesion, were observed to have similar uptake of [^18^F]FGln at baseline and post-CB-839 treatment [35]. In the image analysis, differences in [^18^F]FGln behavior pre- and post-CB-839 treatment were observed only when the data were fit to a reversible 2-tissue compartment model. In this model, only kinetic rate constants k_3_ and k_2_ differed due to treatment. Our data suggest that [^18^F]FGln could still have utility in determining if glutaminolysis is highly utilized in a lung lesion and could be an imaging marker for metastatic progression. Yet, using [^18^F]FGln to monitor CB-839 treatment, at least in lung tissue, might require more sophisticated analysis than simple uptake measurements.

## Conclusion

We demonstrated that [^18^F]FGln could be used to detect the altered metabolism of the UMRC3 cell line *in vivo*. Using a PET/MRI instrument, tumors were easily discerned from normal tissue. This was not possible by PET/CT, where tumor-to-normal tissue contrast was less pronounced. In addition, we observed higher uptake of [^18^F]FGln in ccRCC lines relative to normal kidney cells, which was found to be dependent on ASCT2 expression. Finally, [^18^F]FGln uptake was reduced in the presence of the glutamine transport inhibitor V-9302 and increased in the presence of the glutaminase inhibitor CB-839 in cell culture. However, *in vivo*, we observed no significant change in [^18^F]FGln uptake with CB-839 treatment, highlighting the complex mechanistic nature of this inhibitor. Our results suggest that [^18^F]FGln PET imaging is a promising strategy for ccRCC metastases detection.

## Supplementary Information

Below is the link to the electronic supplementary material.

Supplementary file1 (DOCX 1.56 MB)
