# Supplementary material for: PET/MR Imaging of a Lung Metastasis Model of Clear Cell Renal Cell Carcinoma with (2S,4R)-4-[18F]Fluoroglutamine
Source: Mol Imaging Biol. 2022 Jun 22;24(6):959–72. doi: 10.1007/s11307-022-01747-9 (PMC9681699; doi:10.1007/s11307-022-01747-9)
Supplement: Supplementary file 1 — Supplementary file1 (DOCX 1.56 MB) [file 11307_2022_1747_MOESM1_ESM.docx]

**ELECTRONIC SUPPLEMENTARY MATERIAL**

PET/MR Imaging of a Lung Metastasis Model of Clear Cell Renal Cell Carcinoma with (2*S*,4*R*)-4-[^18^F]Fluoroglutamine

Journal: Molecular Imaging and Biology

Alyssa C Pollard^1,2^, Vincenzo Paolillo^3^, Bhasker Radaram^1^, Sarah Qureshy^1^, Li Li^4^, Tapati Maity^4^, Lei Wang^4^, Md. Nasir Uddin^1^, Christopher G Wood^4^, Jose A Karam^4,5^, Mark D Pagel^1^, David Piwnica-Worms^1^, Steven W Millward^1^, Natalie Wall Fowlkes^6^, William Norton^6^, Brian J Engel^1^, Federica Pisaneschi^1,Ŧ^, Niki M Zacharias^4,Ŧ^

^1^Department of Cancer Systems Imaging, MD Anderson Cancer Center, 1881 East Rd, Houston, TX 77054

^2^Department of Chemistry and Smalley-Curl Institute, Rice University, 6100 Main St, Houston, TX 77005

^3^Cyclotron Radiochemistry Facility, MD Anderson Cancer Center, 1881 East Rd, Houston, TX 77054

^4^Department of Urology, MD Anderson Cancer Center, 1515 Holcombe Blvd, Houston, TX 77030

^5^Department of Veterinary Medicine and Surgery, MD Anderson Cancer Center, 1515 Holcombe Blvd, Houston, TX 77030

^6^Department of Veterinary Medicine and Surgery, MD Anderson Cancer Center, 1515 Holcombe Blvd, Houston, TX 77030

^Ŧ^Corresponding authors (Federica Pisaneschi: fpisaneschi@mdanderson.org, 713-792-5535; Niki M Zacharias: nmzacharias@mdanderson.org, 713-792-5226)

*Radiosynthesis of [^18^F]FGln*

*
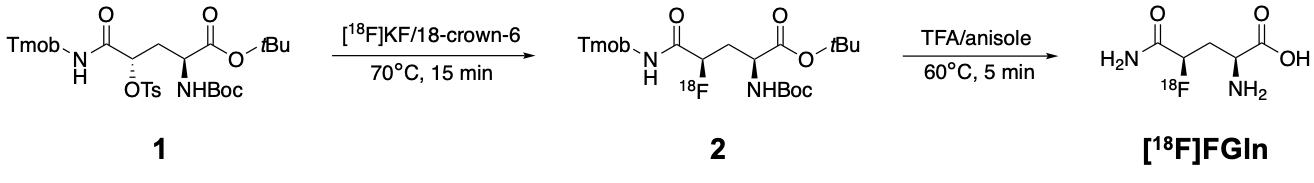
*

**Supplemental Scheme 1.** *Radiosynthesis of [^18^F]FGln.*

Radiosynthesis of [^18^F]FGln was performed using a TRACERlab FX2 N automatic module (General Electric Healthcare, Münster, Germany). [^18^F]Fluoride was obtained as an aqueous solution from the MD Anderson Cyclotron Radiochemistry Facility (CRF). [^18^F]Fluoride was adsorbed on an ion exchange cartridge (pre-conditioned Sep-PAK® Light QMA Cartridge, ABX GmbH, Radeberg, Germany). [^18^F]Fluoride was flushed into reactor 1 with potassium bicarbonate (180 µL of a 8 mg/mL water solution) and 18-crown-6 (8 mg in 1 mL of methanol) contained in Vial 1. The solution was dried under vacuum and a nitrogen flow at 75°C for 4 min. Reactor 1 was cooled to 50°C, and dry acetonitrile (1 mL) was added from Vial 2. The mixture was azeotropically dried at 55°C for 3 min and at 85°C for an additional 3 min. The activity was further dried under vacuum for 3 min.

The synthesis of [^18^F]FGln was carried out in a similar manner reported by Zhang *et al.* [1] by adding the tosylate precursor **1** (5-6 mg) in dry acetonitrile (600 µL) to Reactor 1 through Vial 3 (Supplemental Figure 1a). The mixture was stirred at 70°C for 15 min, then cooled to 45°C. Water (0.6 mL) was added through Vial 4 and transferred to Tube 2. Reactor 1 was rinsed with the high performance liquid chromatography (HPLC) mobile phase (75% methanol/water + 0.1% (v/v) formic acid, 3 mL) via Vial 5, and the solution was transferred to Tube 2. Semipreparative HPLC was performed (Luna 5 µm C18(2) column, 100 Å, 250 x 10 mm), and the radioactive peak (compound **2**) was collected (R*_t_* = 15 min) into a flask prefilled with water (18 mL). The HPLC eluate was loaded into an HLB Oasis cartridge, which was preconditioned subsequently with ethanol (5 mL), air (10 mL), and water (10 mL). After loading the HPLC eluate, the cartridge was washed with water (6 mL, Vial 12) and dried with nitrogen. Radioactivity was then eluted into Reactor 2 with ethanol (1 mL, Vial 13). The ethanol was dried at 60°C, and a trifluoroacetic acid (1 mL)/anisole (10 µL) mixture was added via Vial 7. The mixture was reacted for 5 min at 60°C, then the solvents were removed under nitrogen for 3 min and under vacuum for an additional 1 min. Reactor 2 was cooled to 40°C, and [^18^F]FGln was redissolved in 1 mL of PBS (Vial 8) and transferred into the final delivery vial. Activity was determined with a dose calibrator (Capintec, Mirion Technology, Florham Park, New Jersey, USA), and a sample was taken for quality control (QC). QC was performed by analytical radio-HPLC (Agilent 1260 infinity II, Santa Clara, CA, USA) using a Chirex 3126 (d)-penicillamine chiral column (Phenomenex Inc., Torrance, CA, USA, 1 mM CuSO_4_, 1 mL/min flow rate) (Supplemental Figure 1b). The identity of [^18^F]FGln was confirmed by co-elution with 4-fluoroglutamine, synthesized in house.


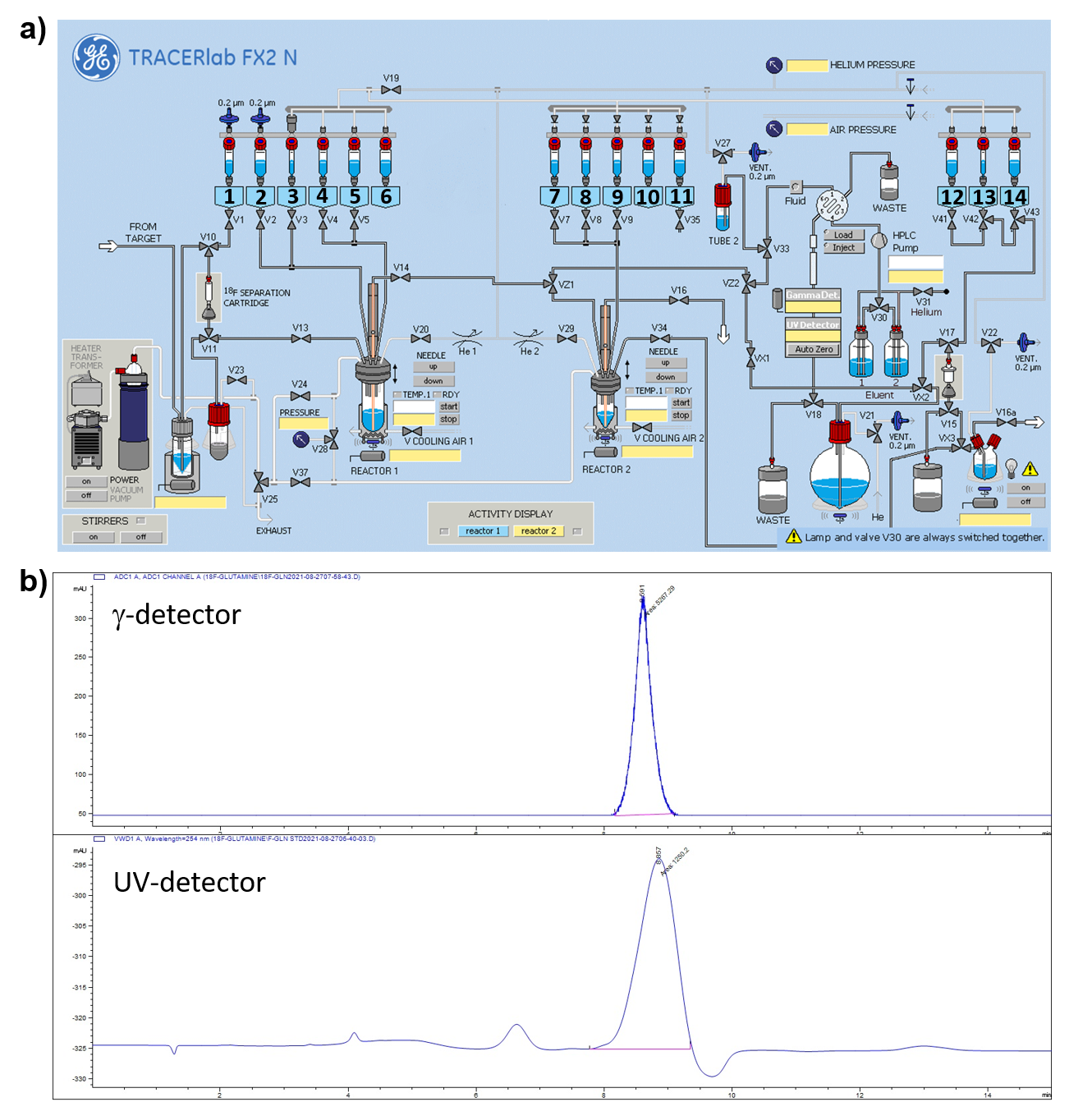


**Supplemental Figure 1.** (**a**) Schematic Diagram of the GE-TRACERlab FX2N. (**b**) QC analysis by radioHPLC of [^18^F]FGln.

*Cell Culture*

The UMRC3-LUC-GFP cell line was generated using viral transfection (adenovirus of a standard GFP-luciferase vector) into the clear cell renal cell carcinoma UMRC3 cell line [2]. For all mouse injections, this cell line and SN12C were grown in Minimum Essential Medium Eagle (MEM, Corning, 10-010-CV, Glendale, AZ) containing 1% (v/v) MEM Nonessential Amino Acids (Corning, 25-025-CI), 1% (v/v) penicillin-streptomycin solution (Corning, 30-002-CI), and supplemented with 10% (v/v) fetal bovine serum (Millipore Sigma Aldrich, F0926, St. Louis, MO). Short tandem repeat analysis was used to authenticate the cell lines. Cells were detached using 0.25% (v/v) trypsin with 2.21 mM EDTA. Trypan blue staining was used to determine cell viability. Cells were counted using a Countess automated cell counter (ThermoFisher, Waltham, MA).

*Bioluminescence Imaging (BLI)*

D-Luciferin sodium salt (Gold Bio, LUCNA-1G, St. Louis, MO) was dissolved in Dulbecco's Phosphate Buffered Saline (DPBS) without calcium and magnesium to a concentration of 15 mg/ml and aliquoted. Aliquots were kept at -20°C until utilized for imaging. 200 µL of luciferin solution were administered by intraperitoneal injection (i.p.). Mice were then imaged using an Xenogen IVIS-200 Optical Imaging System (PerkinElmer, Waltham, MA) at 5, 10, and 15 minutes post-injection using a 3-minute acquisition, 13.5 or 23 cm field of view, and a binning factor of 8. Images were processed using Living Image Software (PerkinElmer, Waltham, MA). ROIs (2 cm diameter) were drawn over BLI signal found in the left lung of mice and in the right corner of the image (outside of the mouse) to determine background signal for each image. BLI signal was reported in photon flux (p/s). For direct lung tissue BLI measurements, mice were injected i.p. with 200 μl of luciferin solution and imaged two minutes post-injection. Mice were then immediately euthanized by cervical dislocation while the animal was under anesthesia (3% isoflurane), and the lungs were removed by dissection. The lungs were then imaged with the IVIS Optical Imaging System, and BLI was again reported in photon flux (p/s).


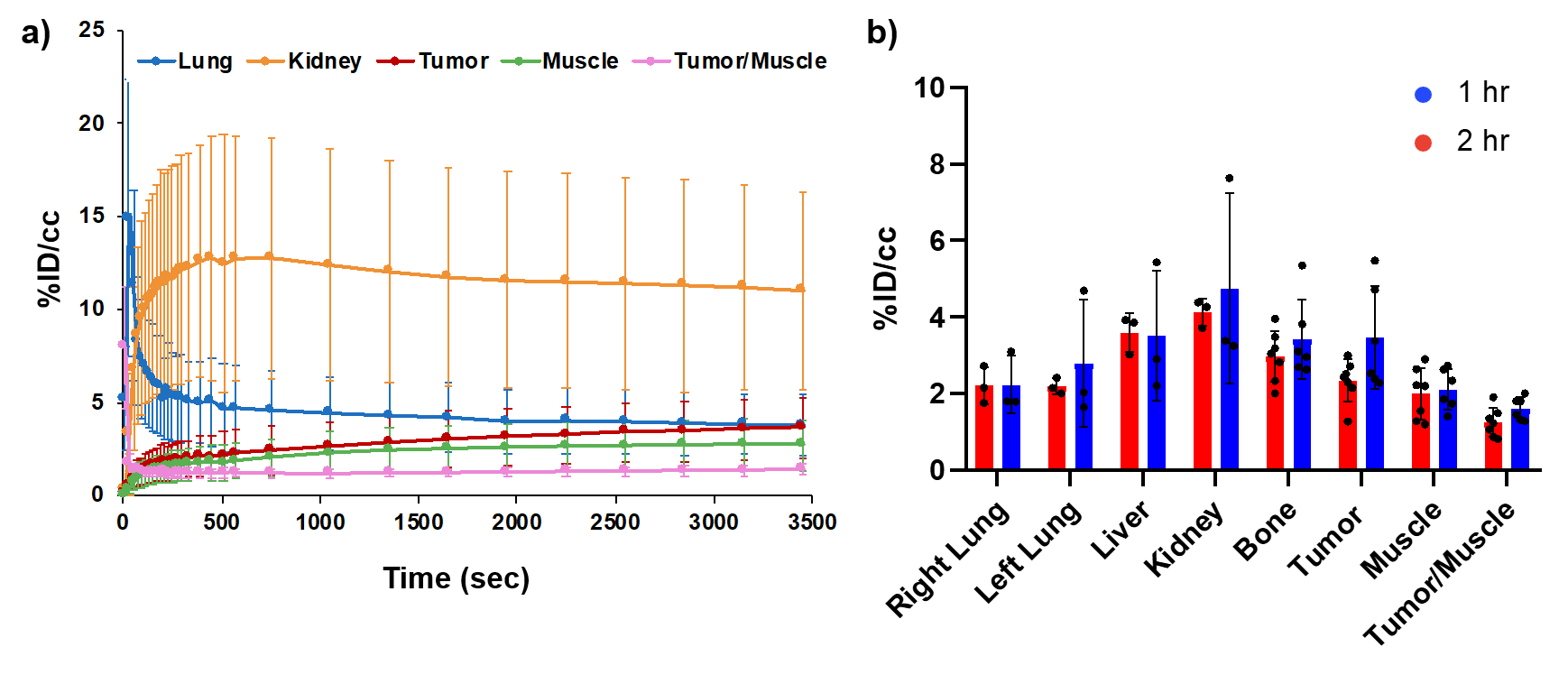


**Supplemental Figure 2.** *[^18^F]FGln biodistribution assays in mice bearing a SN12C subcutaneous tumor.* (**a**) Time-activity curve showed the average uptake of the tracer in the lung, kidney, tumor, and muscle of SN12C subcutaneous tumor-bearing mice (n=4) expressed as %ID/cc. The tumor-to-muscle ratio is also shown in %ID/cc. Error bars represent the standard deviation of the mean. (**b**) For static imaging, mice were imaged in two imaging sessions. Plot of tracer uptake in %ID/cc in the right lung, left lung, liver, kidney, bone, tumor, and muscle of SN12C subcutaneous tumor-bearing mice measured 1 hour (n=7, blue) and 2 hours (n=6, red) post-injection. The tumor-to-muscle ratio is also shown in %ID/cc. Each data point represents an individual mouse; colored columns represent the average; error bars represent the standard deviation of the mean.


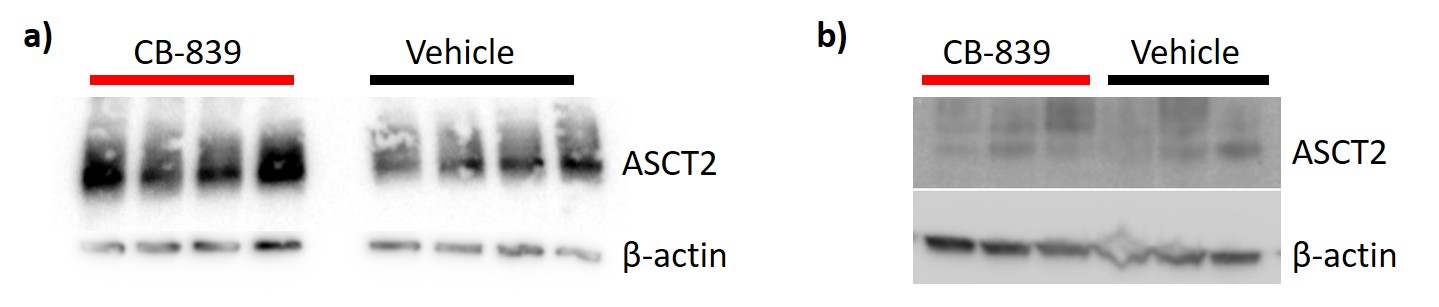


**Supplemental Figure 3.** ASCT2 expression determined by Western blots in vehicle and CB-839 treated UMRC3 cells (**a**) and lung tumors (**b**). (**a**) UMRC3 in culture were treated for 65 hours with 3 μM CB-839 or vehicle (0.01% DMSO), and ASCT2 expression determined by Western blot. (**b**) Fluorescent lung tumor tissue was isolated, flash frozen, homogenized, and lysed. ASCT2 expression was determined by Western blot.


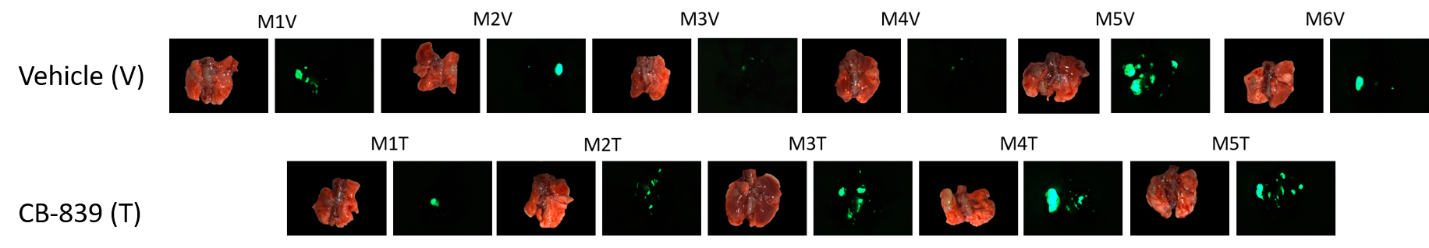


**Supplemental Figure 4**. *Gross pathology and GFP expression in vehicle and CB-839 treated mice.* Brightfield gross pathology of tissue from mice bearing UMRC3 tumors and corresponding fluorescent image of the same tissue. Qualitatively, no difference in GFP signal was seen between vehicle and CB-839 mice.

**References**

1. Zhang X, Basuli F, Shi ZD *et al.* (2016) Automated synthesis of [(18)F(2s,4r)-4-fluoroglutamine on a GE TRACERlab FX-N Pro module. Appl Radiat Isot 110-114.

2. Grossman HB, Wedemeyer G, Ren LQ. (1985) Human renal carcinoma: Characterization of five new cell lines. J Surg Oncol 3: 237-244.
